# Multiscale integration: beyond internalism and externalism

**DOI:** 10.1007/s11229-019-02115-x

**Published:** 2019-02-21

**Authors:** Maxwell J. D. Ramstead, Michael D. Kirchhoff, Axel Constant, Karl J. Friston

**Affiliations:** 1 Department of Philosophy, McGill University, Montreal, QC Canada; 2 Division of Social and Transcultural Psychiatry, Department of Psychiatry, McGill University, Montreal, QC Canada; 3 Wellcome Trust Centre for Neuroimaging, University College London, London, WC1N3BG UK; 4 Department of Philosophy, Faculty of Law, Humanities and the Arts, University of Wollongong, Wollongong, Australia; 5 Amsterdam Brain and Cognition Centre, University of Amsterdam, Science Park 904, 1098 XH Amsterdam, Netherlands

**Keywords:** Boundaries of cognition, Variational free energy principle, Externalism, Internalism, Enactive cognition, Embodied cognition, Markov blankets

## Abstract

We present a multiscale integrationist interpretation of the boundaries of cognitive systems, using the Markov blanket formalism of the variational free energy principle. This interpretation is intended as a corrective for the philosophical debate over internalist and externalist interpretations of cognitive boundaries; we stake out a compromise position. We first survey key principles of new radical (extended, enactive, embodied) views of cognition. We then describe an internalist interpretation premised on the Markov blanket formalism. Having reviewed these accounts, we develop our positive multiscale account. We argue that the statistical seclusion of internal from external states of the system—entailed by the existence of a Markov boundary—can coexist happily with the multiscale integration of the system through its dynamics. Our approach does not privilege any given boundary (whether it be that of the brain, body, or world), nor does it argue that all boundaries are equally prescient. We argue that the relevant boundaries of cognition depend on the level being characterised and the explanatory interests that guide investigation. We approach the issue of how and where to draw the boundaries of cognitive systems through a multiscale ontology of cognitive systems, which offers a multidisciplinary research heuristic for cognitive science.

## Introduction

Over two decades ago, in 1991, Francisco Varela and colleagues articulated a general idea that now underlies what might be called *radical views on cognition*; namely, enactive, embodied, and extended approaches to cognition. According to proponents of the *enactive approach*, “cognition is … the enactment of a world and a mind on the basis of a history of the variety of actions that a being in the world performs” (Varela et al. 1991, p. 9). Since Varela and colleagues, philosophers and scientists have addressed the role of *embodied activity* in cognition and the degree to which our cognitive capacities are realised partly by elements of our embedding environment. Philosophers especially have been considering what embodied, enactive, and extended accounts have to teach us about the *boundaries of cognitive systems*.

Here, we focus on making explicit a description of the boundaries of cognitive systems that we think follows from taking seriously the enactive, embodied, and extended nature of cognition. This is the idea that *the boundaries of cognitive systems are nested and multiple*—*and that, with respect to its study, cognition has no fixed or essential boundaries* (Clark 2017; Kirchhoff 2012, 2018c; Kirchhoff and Kiverstein 2019; Stotz 2010; Sutton 2010).

This idea is far from the accepted view in the philosophy of mind and cognition. Indeed, it is common for researchers from different fields of study—e.g., neuroscience and the philosophy of neuroscience (Hohwy 2014; Seth 2014), embodied cognition (Gallagher 2006; Noë 2004), ecological psychology (Gibson 1979), and anthropology (Ingold 2001)—to infer that there is a *uniquely defining boundary* or unit of analysis from which best to understand and investigate cognition. In its more extreme forms, one might call this position *essentialism* about the boundaries of cognition. Views stressing that cognition has a unique and privileged boundary take many forms. Some argue that cognitive activity is essentially realised by states of the brain. Others argue that cognition is best conceived of as forms of embodied activity. Others still prefer to study cognition “in the wild,” in terms of the patterning of cultural practices and construction of cognitive niches.

The claim that the boundaries of cognition are nested and varied runs counter to any of these brain-based, embodied, and/or ecological, environmental assumptions about the boundaries of cognition, for it does not privilege the brain, the body, or the environment. Nor do we consider the brain, body and environment as equally important, as some in the enactivist tradition have proposed (Hutto and Myin 2013). This is the Equal Partner Principle of radical enactivism. It states that the contributions of the brain to cognition should not be prioritised over those of the body and the environment. Even if there is something correct about this claim—that one should not a priori privilege the brain in explanations of cognition—there is also something problematic about this principle; namely, that on some occasions it will turn out to be incorrect, as privileging the brain will be required to explain some phenomena under consideration.

Where to draw the *scientifically relevant boundaries* will depend both on the nature of the phenomenon being investigated and on our explanatory interests (Clark 2017; Hutchins 1995). By standing on the shoulders of theorists that take seriously the idea that cognitive boundaries are not singular but nested and varied, we reject all views assuming there to be unique and privileged boundaries for cognitive systems, and stake out a compromise position between (in our view) the overly coarse-grained distinction between internalism and externalism about the boundaries of cognition.

Our argument takes the form of a *multiscale integrationist* formulation of the boundaries of cognition based on the variational free energy principle (henceforth FEP). This principle casts cognition and action in terms of quantities that change to minimise free energy expected under action policies. As we discuss in the second section of this paper, we use the FEP because free energy and its expectation can be broadly construed as metrics of cognitive activity that transcend specific spatial and temporal scales (Friston et al. 2015; Kirchhoff 2015; Kirchhoff et al. 2018; Ramstead et al. 2018a, 2019). This allows us to cast the boundaries of cognition as assembled and maintained in an informational dynamics across multiple spatial and temporal scales. Crucially, we shall show that this multiscale application of the FEP implies both ontological and methodological pluralism.

We cast *ontological pluralism* in terms of a *multiscale formal ontology of cognitive systems*. In the sense we are using the term, to produce a *formal ontology* means to use a mathematical formalism to answer the questions traditionally posed by metaphysics; i.e., what does it mean to be a thing that exists, what is existence, etc. Our formal ontology is effectively in the same game as statistical physics, in that it treats as a system sets of states that evince a robust enough form of conditional independence.

This ontology implies, that any given cognitive system has a plurality of boundaries relevant to their scientific study; namely, the boundaries of its relevant subsystems. Our claim is that which among these are the *most relevant* will depend on the phenomenon being studied and the explanatory interests of researchers. Some of these boundaries are internal to the systems—these are boundaries of relevant *subsystems* nested in the whole system or organism (e.g., cells, ensemble of cells, organs, etc.); other boundaries separate the organism from its external environment (e.g., the skin membrane); and others still extend outwards to include the organism and external, worldly states (e.g., constructed niches and patterned cultural practices).

The claims we are making about the boundaries of cognitive systems are ontological. We are using a mathematical formalism to answer questions that are traditionally those of the discipline of ontology, but crucially, we are not deciding any of the ontological questions in an a priori manner. The Markov blankets are a result of the system’s dynamics. In a sense, we are letting the biological systems carve out their own boundaries in applying this formalism. Hence, we are endorsing a dynamic and self-organising ontology of systemic boundaries.

Furthermore, this ontological pluralism implies *methodological pluralism* under the FEP. The FEP can be used as a *methodological heuristic* for interdisciplinary research, which in turn allows scientists to privilege various boundaries of a nested cognitive system, depending on their specific explanatory interests. The FEP is *not* a theory of everything; it does not, on its own, provide an explanation of the systemic processes that constitute living systems (Ramstead et al. 2018b). Rather, it is a principle that coordinates and constrains the kind of explanations deployed when one is addressing how expected free energy minimisation occurs across many different spatial and temporal scales; which call for complementary explanations in terms of, e.g., neuroscience (Friston 2010), embodied cognition (Allen and Friston 2018), ecological psychology (Bruineberg and Rietveld 2014; Ramstead et al. 2019), and niche construction (Constant et al. 2018a; Hesp et al. 2019).^1^

**Fig. 1:**
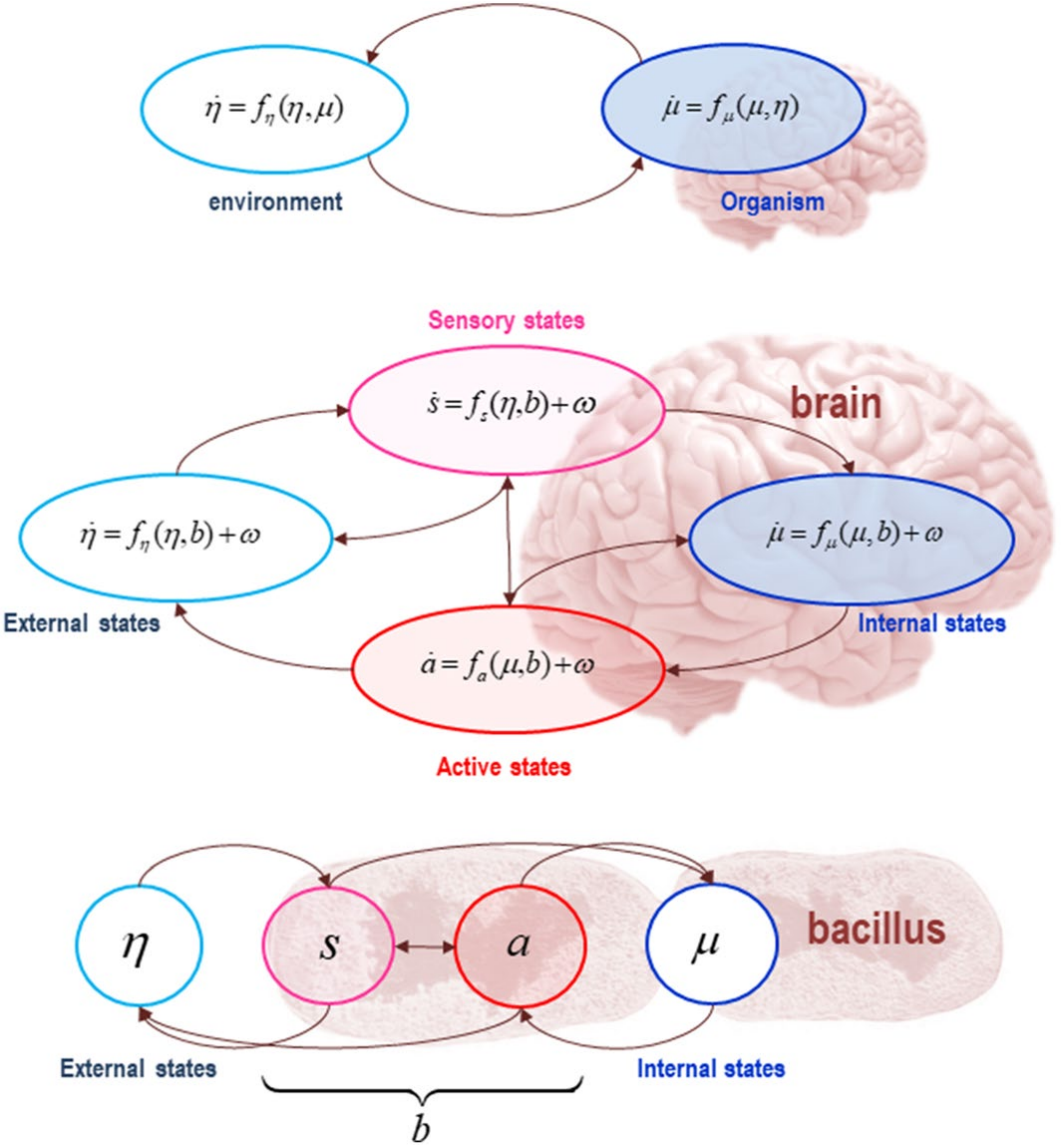
The Markov blanket and active inference. A Markov blanket is a set of states that enshrouds or statistically isolates internal states from external or hidden states. This figure depicts the partition of the states into internal ($\mu$) and external states ($\eta$). In the parlance of graph theory, the Markov blanket is a set of nodes that shields the internal states (or nodes) from the influence of external states; in the sense that internal states can only be affected by external states indirectly, via the blanket states (Friston et al. 2017b). Internal and external states are therefore separated, in a statistical sense, by the Markov blanket ($b$), which itself comprises sensory ($s$) and active states ($a$)—defined as blanket states that are and are not influenced by external states respectively. The top panel schematises the relations of reciprocal causation that couple the organism to its ecological niche, and back again. Internal states of the organism change as a function of its current state ($\mu$) and the state of its niche ($\eta$), which is expressed in terms of a flow $f_{\mu } (\mu ,\eta )$ with random fluctuations. Reciprocally, states of the niche change over time as a function of the current state of the environment and the organism, again, specified in terms of a flow $f_{\eta } (\eta ,\mu )$ with random fluctuations. The self-organisation of internal states in this scheme corresponds to perception. Active states couple internal states back to states of the niche, and so correspond to the actions of an organism. Given the anti-symmetric conditional dependencies entailed by the presence of the Markov blanket, the dynamics of the niche, too, can be expressed as a gradient flow of a free energy functional of external and blanket states. The lower panel depicts the dependencies as they would apply to a unicellular organism. In this panel, the internal states are associated with the intracellular states of a cell, the sensory states are associated with surface states of the cell membrane, and the active states are associated with the actin filaments of the cytoskeleton. Adapted from Constant et al. (2018b)

We approach this multiscale, integrationist view of the boundaries of cognition by focusing on the *Markov blanket formalism*, which underwrites the FEP (see Fig. 1 for a detailed technical explanation). This formalism allows us to individuate a system by demarcating its boundaries in a statistical sense. Intuitively, for a thing to exist, it must evince some form of conditional independence from the system in which it is embedded. Markov blankets operationalise this intuition. In more technical terms, a Markov blanket induces a statistical partitioning between internal (systemic) and external (environmental) states, where environmental states can be associated with neuronal, bodily, or worldly states depending on the relevant partitioning of the system in question. The Markov blanket itself comprises a bipartition into active and sensory states, which mediate exchanges between systemic and environmental (neuronal, bodily, worldly) states. Importantly, the presence of a Markov blanket shields or insulates internal states from the direct influence of external states. This follows from the partitioning rule of Markov blankets, according to which internal states can influence external states via active states, and external states can influence internal states via sensory states. Hence, the Markov blanket formalism shows that internal and external states are ‘hidden’ (i.e., conditionally independent) from one another in virtue of the existence of a Markov blanket, thus providing the statistical means by which to delineate the boundaries of a biological and/or cognitive system.

We accept that the Markov blanket formalism can be used to delineate the boundaries of cognitive systems (cf. Hohwy 2016; Kirchhoff and Kiverstein 2019). We shall argue that cognition involves dynamics (i.e., the Bayesian mechanics of active inference) that ensure adaptiveness, which straddle across and integrate such boundaries. We call this position *multiscale integration*. We argue that the FEP can accommodate a multiscale integrationist account of the boundaries of cognitive systems. We therefore argue that the *inferential seclusion* of internal states and external states, given by the Markov blanket formalism, can coexist with *existential integration* through active inference; justifying the view that the boundaries of cognition are *nested* and *multiple*.

The structure of this paper is as follows. In the next (second) section, we review the FEP and active inference. In the third section, we survey key principles of new radical—extended, enactive, embodied—views of cognition, with a focus on enactive views in particular. We then describe a brain-based argument for the boundary of cognitive systems premised on the Markov blanket formalism—and the FEP—that pushes back again these radical views of cognition. In the fourth section, we develop our positive proposal for a multiscale account of the FEP. We argue that the encapsulation or statistical seclusion entailed by the Markov boundary is reiterated at every hierarchical description of living systems; from the single cell, to organs, to individuals, and all the way out to coupled organism-environment systems—all of which can be cast as having their own Markov blanket. We also argue that the organism and niche are coupled to one another through active inference.

In this sense, our argument owes much to (Clark 2017). Clark sets out the idea of organisms having temporally extended Markov blankets, the boundaries of which reach all the way down to DNA and all the way up to individual organisms and their respective niches. Our focus, however, is different from Clark’s, in two ways. First, we make explicit that this view of the Markov blanketed cognitive system implies two forms of pluralism, ontological and methodological; and second, we emphasize that active inference entails adaptive phenotypes, cultural practices, and niche construction; the joint phenotype of the organism (including states of its adapted niche) encodes information that, at least in some cases, is as important as that encoded by states of the brain to explain adaptive behaviour. We conclude by considering future research directions for approaching systemic organisation through a multiscale ontology of cognitive systems and a multidisciplinary research heuristic for cognitive science.

## A variational principle for living systems

### The variational free energy formulation

Organisms find themselves, more often than not, in a bounded set of characteristic states. We can cast this set of states, in which the organism is *most likely* to find itself, as its overall *phenotypical states and traits*; namely, the repertoire of measurable functional and physiological states, as well as morphological traits, behavioural patterns, and the adapted ecological niches that characterizes it as ‘the kind of organism that it is’ (Kirchhoff et al. 2018; Ramstead et al. 2018a). From this statistical perspective, the question of how organisms remain alive can be recast as the question of how they maintain themselves in phenotypic states.

Remarkably, organisms resist entropic erosion by simply limiting the dispersion of states that they occupy during their lifetime. The variational free energy principle (FEP) provides a formal description of this anti-entropic feat. The FEP casts the functioning of biological systems of any kind, including their different psychological profiles, in terms of a single imperative: to minimise *surprise* (aka surprisal or self-information). The concept of surprise does not refer to the psychological phenomenon of being surprised. It is an information-theoretic notion that measures how uncharacteristic or unexpected a particular sensory state is, where *sensory* states can be caused by *external* worldly (and bodily) states.

**Fig. 2:**
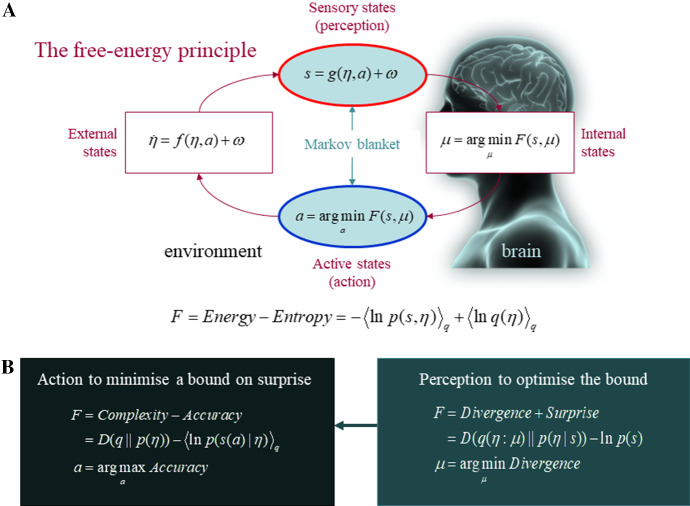
The free energy principle and self-evidencing. Upper panel: depiction of the quantities that define an agent engaging in active inference and its coupling to its ecological niche or environment. These are the internal states of the agent ($\mu$), sensory input $s = g(\eta ,a) + \omega$, and action $a$. Action and sensory input describe exchanges between the agent and its world; in particular, action changes how the organism samples its environment. The environment is described by equations of motion, $\dot{\eta } = f(\eta ,a) + \omega$, that specify the (stochastic) dynamics of (hidden) states of the world $\eta$. Here, $\omega$ denote random fluctuations. The free energy ($F$) is a function of sensory input and a probabilistic belief $q(\eta :\mu )$ that is encoded by internal states. Changes in active states and internal states both minimise free-energy and, implicitly, self-information. Lower panel: depiction of alternative expressions for the variational free-energy, which clarify what its minimisation entails. With regards to action, free-energy can only be minimised by increasing the *accuracy* of sensory data (i.e., the selective sampling of predicted data). Conversely, the optimisation of internal states through perception makes the probability distribution encoded by internal states an approximate conditional density on the causes of sensory input (by minimising a Kullback–Leibler divergence $D$ between the approximate and true posterior density). This optimisation tightens the free-energy bound on self-information and enables the creature to avoid surprising sensations through adaptive action (because the divergence can never be less than zero). With regards to the selection of actions that minimise the *expected free energy*, the expected divergence becomes (negative) *epistemic value* or *salience*, and the expected surprise becomes (negative) *extrinsic value*; which is the expected likelihood that prior preferences are indeed realised as a result of the selected action. See (Friston et al. 2017) for a full description of the free energy expected following an action. Adapted from Ramstead et al. (2018a)

A key premise of the FEP is that cognitive systems cannot estimate surprise directly and therefore must work to reduce an *upper bound* on surprise, which they can track; namely, variational free energy. In other words, surprise cannot be evaluated directly because this would entail to name all possible ways in which some sensations could have been caused. However, variational free energy can be evaluated given a generative model of how sensations were caused. Because variational free energy is (by construction) always greater than surprise, minimising free energy implicitly minimises surprise (see Fig. 2). One can think of variational free energy as a *guess* or *approximation to surprise*, whose accuracy can be finessed through perception; namely, the dynamics of a system’s internal states. This perceptually crafted approximation to surprise can now be minimised by action; namely, the dynamics of a system’s active states.

In a nutshell, the FEP tells us that cognitive systems can estimate and thereby avoid surprise, on average and over time, by working to suppress a variational bound on surprise. Crucially, this free energy bound is exactly the same quantity used in Bayesian statistics to optimise (generative) models of data. In this setting, negative free energy is known as log model evidence or marginal likelihood. This leads to a complementary perspective on surprise-minimising dynamics that become self-evidencing; in the sense of optimising Bayesian model evidence—and, by implication, performing some sort of (perceptual) inference. In short, technically speaking, minimising self-information underwrites self-organisation through self-evidencing (Hohwy 2016); thereby evincing a Bayesian mechanics for any system that exists in the sense of possessing a Markov blanket.

Standard cognitive functions like perception (Hohwy et al. 2008), attention (Feldman and Friston 2010), and learning (Friston 2005; Friston et al. 2016a) all seem to conform to this single principle. The machinery used to estimate and avoid surprise also recruits a series of non-standard functions like emotions (Van de Cruys and Wagemans 2011), action (Friston et al. 2011), culture and its production (Fabry 2018; Ramstead et al. 2016), as well as evolutionary processes like niche construction (Constant et al. 2018a, b) and natural selection (Campbell 2016; Friston and Stephan 2007), thereby forcing us to rethink the boundaries of cognition.

Statistically, one can define variational free energy as surprise plus a measure of the distance between a system’s (posterior Bayesian) beliefs^2^ about the external causes of its sensory input, encoded by its internal states (e.g., neural architecture), and the true posterior probability distribution, conditioned on a generative model of how that input was produced (Friston 2010). Thus, the variational free energy is defined with reference to a (generative) model of what caused its sensations (including, crucially, its own actions). Variational free energy can thus be cast as a measure of the kinds of things that the cognitive systems finds surprising or, more simply, an estimation of surprise. In summary, variational free energy is an *upper bound* on surprise, in the sense that surprise can never be greater than free energy given the way variational free energy is constructed—for details, see Friston (2012). Thus, by acting to minimise free energy, organisms implicitly minimise surprise.

Crucially, by acting to reduce variational free energy, biological systems come to instantiate a *probabilistic* (*generative*) *model* of their environment, including the states of their body (Friston et al. 2017b). This generative model can be viewed as a ‘map’ of the relational or causal structure among the various quantities (e.g., sensory observations and Bayesian beliefs) that are optimized through action, perception, and learning, as the organism navigates, and maintains itself in its environment. Hence, it is said that the generative model is ‘entailed’ by the existence of an organism (Friston 2012; Ramstead et al. 2018a), in the sense that it changes as a function of the organism’s normal bioregulatory activity. Heuristically, this means that through adaptive action, organisms come to embody a guess about the causes of their sensations (i.e., a generative model) by optimizing its beliefs about those causes.

An intuitive example of free energy bounding dynamics is the maintenance of core body temperature. Human beings tend to maintain their body temperature around 36.5 °C. Human bodies expect to be in typical (phenotypical or characteristic) states; surprise is large if the probability of the sensory state is low. So, any deviation from the mean, 36.5 °C, implies that the organism is in a sensory state with (relatively) high surprise. Conversely, surprise is low when the probability of the sensory observation is high. Importantly, deviations from the expected (i.e., the mean) state induce *active inference*.

Active inference refers to the joint optimisation of internal states (e.g., perception) and the selection of action policies (i.e., sequences of active states that minimize expected free energy), which function hand-in-hand to reduce free energy (resp. surprise). The system of nested subsystems reacts as a whole, at various scales, to discrepancies between the predictions under the generative model and the actual state of the world. Active inference can take many forms in this setting. Reactions to departures from expected temperature include, at one scale, individual reactions from temperature-sensitive sensory cells in the skin; the raising of individual hairs by skin cells; the registering of a temperature difference by the networks of the nervous system, and the body’s subsequent engaging in shivering behaviour. More individual, psychological reactions to changes in temperature might include enjoying this change (or not); culturally-mediated behavioural reactions to differences in temperature might come into play as well, relying on elements of the cultural niche. If it is too hot, we might take off some clothes; but if one lives in the desert, this exposes one’s bare skin to the elements; and to fend off the heat, we might instead put on robes, as Bedouins do in the desert.

### Generative models and action policies

In the variational approach, the form taken by the generative models is that of *graphical models* (Friston et al. 2017b). The model itself carries *correlational information about causal factors that lead to the generation of sensory states*. So, in a nutshell, the model is *intrinsically* probabilistic and correlational, not causal; in the sense that the generative model, by necessity, captures useful probabilistic information about the agent acting in its niche.

Technically, the generative model is just a probability distribution over the joint occurrence of sensory states (of the Markov blanket) and the external states generating sensory states. It is a *normative* model, in the sense that it specifies the conditions that allow the continued existence of the type of creature being considered. This can be variously formulated in terms of the likelihood of some sensory states, given external states and prior beliefs over external states. It manifests in active inference via inferential dynamics (i.e., action and perception) that flow on free energy gradients, where the free energy is defined in terms of a generative model.

However, the variational story is one about how the respective statistical structures of the generative model and generative process (the actual causal structure that generated observations) become attuned to one another. So, when everything is going well (i.e., when the organism engages in adaptive behaviour and thrives in its niche), the correlational structure carried by the generative model—ideally—maps onto the causal structure of the generative process in the environment. So, while the model is necessarily only ever probabilistic, it remains that active inference fits or tunes the generative model to the generative process; and by that fact, the generative model gains some causal purchase: indeed, the generative model is often described as a probabilistic description of how sensory consequences are generated from their causes. Inference then corresponds to the inversion of this mapping—to infer causes from consequences. This is inference is, by construction, implicit in the minimisation of free energy or the maximisation of model evidence.

One novel way to think about the generative model is in terms of ‘enactment’. On this view, minimising free energy essentially means reducing the disattunement between the expectations of an organism and the generative model under which actions are selected (Bruineberg and Rietveld 2014). Active inference is the process of creating and maintaining self-organization through action. Under the FEP, active sampling of sensory states is a feature of the entire dynamics themselves, which entail a generative model. This speaks to the idea that the entire process of attuning the system to its niche involves perceptual inference, but especially the selection and expression of relevant *action policies*—policies that select the actions most likely to elude surprise. Minimising expected surprise does not mean avoiding sensations, on the contrary, it means resolving uncertainty by seeking out salient, informative sensations. This follows simply from the fact that *expected surprise* (i.e., self-information) corresponds to *uncertainty* (i.e., entropy) (Friston et al. 2016b; Friston et al. 2018)

This implies that the function of the generative model is to guide action in a context-sensitive fashion; in turn, this speaks to a shift away from viewing the brain in terms of Bayesian predictive processing to how the brain enables “feedback loops that maintain attunement to the environment and support adaptive behavior” (Anderson 2017, p. 8). This dynamic emphasis on the realisation of biological self-organisation through adaptive action clearly aligns the FEP with enactive and pragmatist approaches to cognition (Bruineberg et al. 2016; Engel et al. 2016; Kirchhoff and Froese 2017; Ramstead et al. 2019)—a point we will explore in greater detail in Sect. 4.

### Markov blankets and the boundaries of cognitive systems

Under the FEP, the statistical conception of life leads to a formal, statistical ontology of living systems (Friston 2013; Kirchhoff et al. 2018; Ramstead et al. 2018a). This ontology leverages a statistical formalism; namely, the Markov blanket formalism, which provides a principled account of what constitutes a system, and what does not. A Markov blanket is a statistical partitioning of a system into internal states and external (i.e., non-constitutive) states; where the blanket itself can be partitioned further into sensory and active states (Clark 2017; Friston 2013; Pearl 1988). This implies that internal and external states are conditionally independent from one another, given that internal and external states can only influence each other via sensory and active states.

A Markov blanket constitutes the evidential or existential boundary that sets something apart from that which it is not. A cell therefore has a Markov blanket—its plasmalemma. As do multicellular organisms like *Homo Sapiens*. Take the cell as an example. It arises out of a molecular soup by assembling its own boundaries, thus acquiring an identity (Friston 2013; Varela et al. 1991). For a cell to remain alive, its internal states must constantly organise and prepare its boundaries—lest it decay, and dissipate into its surroundings (Di Paolo 2009).

This, in turn, implies the maintenance of a statistical boundary that separates internal from external states, and vice versa (Friston 2013). Under the FEP, this statistical boundary is an *achievement*, rather than a given; it is generated and maintained through active inference (i.e., adaptive action). This again aligns the FEP with enactive and pragmatist approaches to cognition (Engel et al. 2016). Thus, under the FEP, to exist ‘just is’ maintaining the states that comprises one’s Markov blanket through active inference. In other words, without a Markov blanket and the processes that assemble it, the cell would cease to exist, as there would be no way for the cell to restrict itself to a characteristic set of states. In other words, there would be no way of establishing the conditional independence between internal states and the surrounding environment—and the cell would simply dissolve, dissipate or decay into its universe (Hohwy 2016).

The nice thing about Markov blankets is that they allow us to speak in a meaningful (and mathematically tractable) way about conditional independencies between internal and external states. Consider again the cell. The intracellular (i.e., internal) states of a cell have an existence that is distinct from their external environment. This shows that intracellular and extracellular states are conditionally independent. It is the conditional independence (in a statistical sense) between internal and external states that are captured—or indeed defined—by appeal to the concept of a Markov blanket (see Fig. 1).

### A formal ontology for the boundaries of cognitive systems

This reading of active inference as self-evidencing makes the boundary of cognitive systems an *existential* notion, tied up with the epistemic process of generating evidence for your own existence. In a nutshell, then, to enact a generative model is to provide evidence (i.e., to generate evidence through adaptive action) for a model of one’s existence.

More specifically, the claim we are making about the status of the boundary of cognitive systems is that *this boundary is both ontological and epistemological*. The boundary of a given cognitive system is given by the Markov blanket of that system, which carves out or individuates a system by separating systemic states from non-systemic ones. The Markov blanket is an *ontological* boundary, in the sense that this boundary individuates the system as the kind of system that it is. It sets apart the states that count as systemic states from those that count as part of its surroundings. Markov blankets provide the most minimalistic answer to this question, based on the notion of *conditional independence*. If a system exists, there must a sense in which the non-systemic parts can change without the system of interest changing in concert. Markov blankets formalise this requirement. The Markov blanket is a result of the system’s dynamics (i.e., the system’s patterns of adaptive action), which means that it is the system’s dynamics itself that carves out the relevant boundaries. In other words, the boundary itself is orchestrated and maintained through active inference, it is an achievement of the cognitive system that is orchestrated and maintained through adaptive action.

We claim that the Markov blanket is an *epistemological* boundary as well. This is because the boundary is realised through active inference, which is a process of *self*-*evidencing*. Self-evidencing means that *to exist as a system* is to produce *evidence of your existence*. More explicitly, the variational framework suggests that the dynamics of living systems entails a *generative model* of one’s existence. The variational framework tells us how the generative model that organisms embody and enact tunes itself to (approximates the statistical structure of) the generative process, or actual causal process in the environment that causes the sensory states of an organism. To exist as a living being and to engage in adaptive action (when all goes well) *just is* to realise the relations between quantities that are modelled in the generative model. In other words, under the FEP, to exist at all means to produce evidence for a model of oneself (or more exactly, since the generative model is a control system, a model of oneself acting in the world).^3^ Existence in this sense is fundamentally tied up with the creation and maintenance of an informational boundary, i.e. the Markov blanket.

The Markov blanket formalism, then, tells us what counts as a system and what does not. It provides us with a principled means to determine what it is to be a *self*-*evidencing system* under the FEP. In this sense, the term *existential* boundary might be most appropriate: the evidential boundary is also an existential boundary.

In summary, when applied to the biological realm, the statistical formalism of the Markov blanket provides a way to define the boundaries of a system. To so enshroud the internal (constitutive or insular) states of a system behind a Markov blanket enables the individuation of a well-defined partition of the system into internal and external states, mediated by the (active and sensory) states that comprise the Markov blanket itself, and over which we can define systemic dynamics.

## Cognitive boundaries: externalism and internalism

In this section we have two agendas. The first is to address externalist or radical views of cognition; namely, embodied, enactive, and extended cognition. We will pay special attention to enactive formulations of life and mind, highlighting that on this account, the basis of life and mind is a nested set of properties: autopoiesis, operational closure, autonomy, and adaptivity. The nice thing about this formulation of living and cognitive systems is that it allows us both to address the organisational principles of life, as per the enactive framework, and speak to how this framework underpins the ideas of cognition as realised across brain, body, and world; while, at the same time, giving a special place to embodied activity in the assembly of cognitive activities and processes. Our second agenda is to describe how this emphasis on (especially) adaptive operational closure could be turned into an argument against the enactive view by appeal to the active inference scheme and the Markov blanket formalism.

### Externalism: radical views of cognition

Embodied approaches to cognition hold that the body is crucial for cognition (Gallagher 2006). Extended views suggest that not only are bodies important, the local environment of individual cognitive systems can partly realise cognitive processes (Clark 2008; Clark and Chalmers 1998). Enactive views play up the role of action in the functioning of cognition, especially on certain accounts of enactivism tethering mind to the biology of living systems (Chemero 2009; Gallagher 2017; Thompson 2010). In this subsection we formulate the enactive view associated with the work of Varela and colleagues; so-called autopoietic enactivism (Di Paolo 2009; Di Paolo and Thompson 2014; Thompson 2010; Varela et al. 1991). Our focus is selective; the enactive framework not only exemplifies current radical views on cognition, it also shares a number of important overlaps with our multiscale integrationist view, derived from the FEP.

**Fig. 3:**
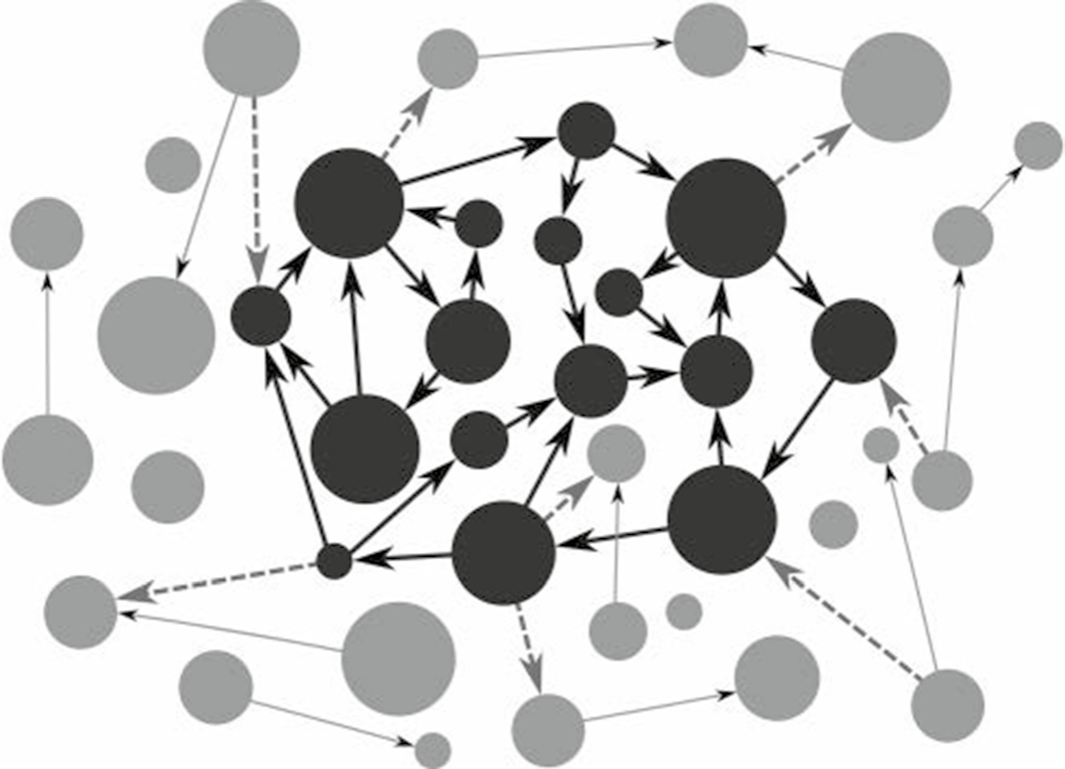
An illustration of operational closure. Here the black circles form part of an operationally closed network of self-organising processes. Each black circle has at least one arrow arriving at it and at least one arrow coming from it—respectively originating and ending in another black circle. Dashed arrows refer to enabling relations between processes in the operationally closed network and processes that do not belong to it. Adapted from Di Paolo and Thompson (2014, p. 70)

A central aspect of living and cognitive systems is their *individuation*. Individuation is the process that makes something distinct from something else, and is in this sense consistent with our use of the Markov blanket formalism as a means by which to delineate systemic boundaries separating systemic from non-systemic states, and vice versa. Crucially, on the enactive account, this process of individuation implies that systems that can self-organise their own process of individuation are (a) *autopoietic*, (b) *operationally closed*, and (c) *autonomous*. Autopoiesis denotes the property of structural self-generation; namely, the capacities to (re-)generate and maintain systemic constituents, despite compositional and functional change. An autopoietic system can be cast as an operationally closed system. Operational closure refers to processes of autopoietic self-assembly, on the one hand, and boundary conservation conditioned on interdependent processes, on the other. This is entirely consistent with the kind of statistical independence between states induced by the Markov blanket formalism, as this implies that the very existence of a living system is premised on recurrent processes that work to conserve the integrity of systemic boundaries (see Fig. 3).

**Fig. 4:**
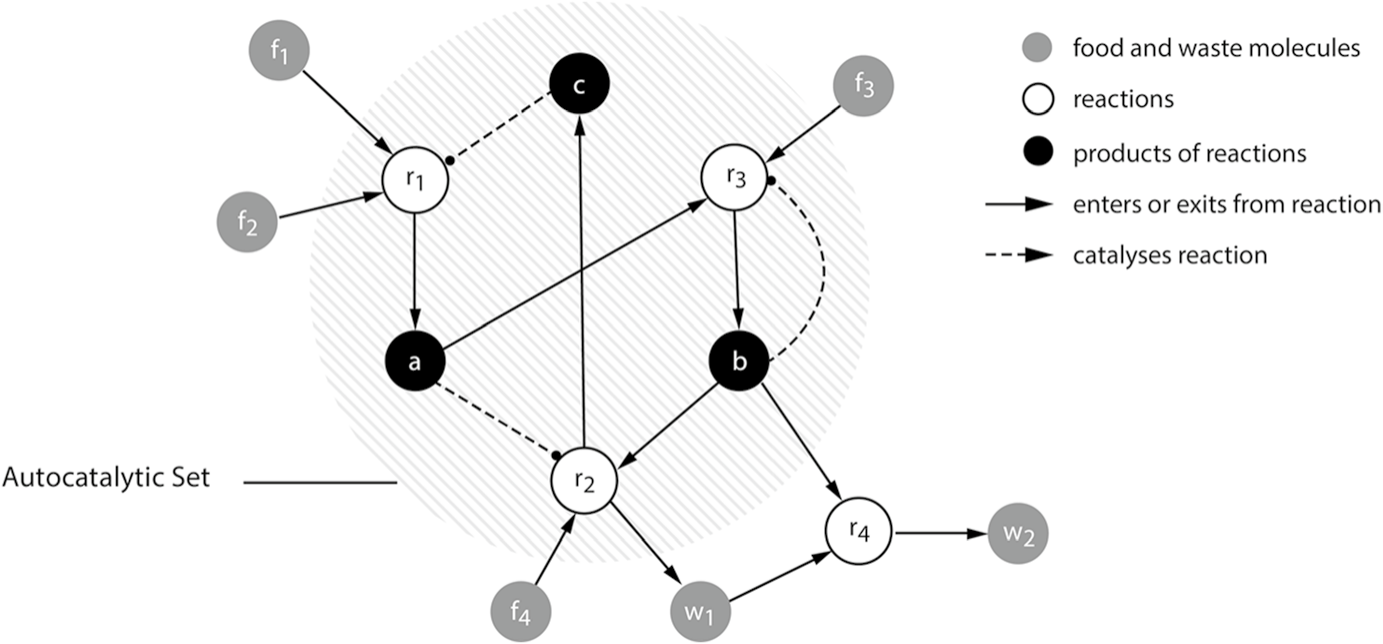
Schematic illustration of autocatalytic closure. A network of chemical reactions is organized such that each reaction is enabled or catalyzed by products of other reactions in the network. From Di Paolo et al. (2017, p. 113)

In an operationally closed network each process is affected by another process such that the operations of processes comprising the network are dependent on each other. As Di Paolo and Thompson put it, in relation to this figure: “If we look at any process in black, we observe that it has some enabling arrows arriving at it that originate in other processes in black, and moreover, that it has some enabling arrows coming out of it that end up also in other processes in black. When this condition is met, the black processes form a network of enabling relations; this network property is what we mean by operational closure.” (Di Paolo and Thompson 2014, p. 71). To make this a little more concrete, consider Fig. 4.

This figure describes a network of four reactions, *r*_1_, *r*_2_, *r*_3_, and *r*_4_, each of which is enabled—in the sense of being accelerated to sufficiently fast rates—by the molecules of type, *a*, *b*, and *c*, which are themselves the products of the same reactions (Di Paolo et al. 2017). This is an example of an operationally closed network, given that—as a whole—the set is able to enable its own production.

Given what we have said in Sect. 2, it is fairly straightforward to establish that the Markov blanket formalism provides a statistical formulation of operational closure (Kirchhoff et al. 2018). In the same way that active and sensory states of Markov blankets couple internal and external states—via an informational dynamics—operational closure does not imply that the systemic (i.e., operationally enclosed) states are cut-off from external states. To see this, note that autonomy implies that an operationally closed network of self-enabling processes can modulate its relation to the embedding environment. If this were not so, the network would stop or run down. The nice thing about the emphasis on autonomy is that it speaks directly to *adaptivity*, the basic capacity to act purposefully and probabilistically, as the basis of the self-organisation of life and cognition (Di Paolo 2005). In the context of the FEP, this is called *adaptive active inference* (Kirchhoff 2018a, b).

This enactive view of living and cognitive systems exemplifies a radical view of cognition; i.e., a view that breaks faith with the standard assumptions about internalism. First, autopoietic enactivism is a denial of any kind of internalism, given that it is entirely possible for operationally closed dynamics to be realised in an extensive network of processes breaking across neural and non-neural variables (De Jaegher and Di Paolo 2007). Second, autopoietic enactivism denies what is a usual starting point of so-called first-wave or functionalist arguments for the extended mind thesis. First-wave arguments starts by taking the individual as the default cogniser and only then asks whether some worldly elements can play functionally similar roles to mental states or cognitive processes realised internally (Clark and Chalmers 1998). So these arguments for the extended mind assume a kind of internalism in their formulation (for similar critiques, see (Kirchhoff 2012; Menary 2010). Finally, autopoietic enactivism holds the view that cognition is a relational phenomenon between an organism and its environment (Thompson and Stapleton 2009).

### Internalism: pushing back

Despite its influence in the sciences of life and mind, the enactive approach can be put under pressure. Indeed, a specific formulation of the FEP, turning on the Markov blanket formalism, arguably pushed back against any of these radical views of cognition (Hohwy 2016, 2017). Our aim in this subsection is to (briefly) rehearse some of the main steps of this internalist argument. We develop a counterargument to the internalist position in the next section, which gives an information-theoretic justification for the view that the boundaries of cognition are nested and multiple.

In a nutshell, the internalist argument states that the relevant boundary for cognitive systems and cognition is essentially the boundary of the brain or skull. Internalists take the inferential seclusion of internal states in active inference—that internal (systemic) states are hidden behind the veil of the statistical Markov blanket—to imply that the boundaries of cognition stop at the boundaries of the brain given the presence of a brain-bound Markov blanket (Hohwy 2016; Seth 2014).

A crucial aspect of this argument is the assumption that the brain itself is a generative model of its environment, one that “garners evidence for itself by explaining away sensory input” (Hohwy 2017, p. 1) by a process of variational Bayesian inference.^4^ This means that through active inference, a cognitive system minimises its variational free energy, thereby securing the evidence for its generative model, and inferring the hidden causes of its observations (sensory data). Cognitive processes (e.g., attention, learning, decision, perception, and so on) are processes that work to optimise internal states in accordance with the FEP—and implicit self-evidencing (Hohwy 2016).

The next, crucial step is the statistical partitioning of a system into internal and external states through the Markov blanket formalism. This captures the notion that internal (neural) and external (environmental) states are conditionally independent; capable of influencing one another only via sensory and active states. Internalists interpret the Markov blanket as enforcing an evidentiary boundary severing, in an epistemic and causal sense, the brain from its body and environment. Thus, Hohwy concludes: the “mind begins where sensory input is delivered through exteroceptive, proprioceptive and interoceptive receptors and ends where proprioceptive predictions are delivered, mainly in the spinal cord.” (Hohwy 2016, p. 276).

The issue of *internalism* comes up when these two notions, the Markov blanket and active inference, are combined in the free energy formulation. Proponents of internalist readings of the FEP argue that the presence of a Markov blanket implies that systems that minimise their free energy, on average and over time, ipso facto, are epistemically and causally secluded from their environment. The upshot of such a conception is that the boundaries of cognition stop at the skull. Mind is a skull-bound phenomenon. The rationale for this way of thinking is that the spontaneous formation of Markov ensembles realizes a form of Bayesian inference (active inference). Active inference carves out coherent neural ensembles, which are neural ensembles (Hebbian assemblies) ‘wrapped’ in a Markov blanket (Hohwy 2016; Yufik and Friston 2016). This means that cognition implies the transient assembly of such brain-bound Markovian ensembles.

This internalist rendition of internal states, hidden behind of curtains of the Markov blanket, leads to a neo-Kantian or Helmholtzian account of cognition that emphasises its indirect nature (Anderson 2017; Bruineberg et al. 2016). The Markov ensembles are said to infer external states, and this inference is taken to be a content-involving affair. This means that inferences are over content-involving states (in the sense that internal states that are *about* things in the world), which are cast as hypotheses and beliefs.^5^ The idea is that organisms leverage their generative model to infer the most likely hidden causes of its sensory states. This is a Helmholtzian interpretation of the FEP (Bruineberg et al. 2016). On this reading, active inference is understood on an analogy with scientific inference, as literal hypothesis-testing.

This kind of neo-Kantian schism between mind and world is taken to imply that the contact of a cognitive system with its environment—perceptually or behaviourally—is mediated by its internal (neural) states, often interpreted as representations encoded in hierarchical generative models that are realised in the brain’s cortical architecture (Gładziejewski 2016; Gładziejewski and Miłkowski 2017; Williams 2018). We shall not dwell on the question whether these internal states are representations—for contrasting interpretations, see (Kirchhoff and Robertson 2018) versus (Kiefer and Hohwy 2018). However, in the next section, we consider whether internalist interpretations of Markov blankets and generative models are appropriate.

## Multiscale integration: nested and multiple boundaries

In this section, we argue that the internalist interpretation of the boundaries of cognition rests on a problematic interpretation of what generative models are, and the kind of properties they have under the FEP. Crucially, we agree with internalism that any relevant mind–world relation is mediated by processes that can be cast as both assembling and finessing the generative model. But this is just to say that we can describe how internal and external states are statistically coupled to one another via intricate and complex sensorimotor dynamics (Gallagher and Allen 2016; Kirchhoff and Froese 2017).

### Generative models: what they are, and how they are used to study cognition

First, we take issue with the claim that, under the FEP, the generative model is something internal to the organism (i.e., that the generative model comprises neuronal vehicles or any other vehicles). Rather, the generative model is a mathematical construct that explains how the quantities embodied by the system’s architecture change to transcribe (i.e., update beliefs about) the causes of the system’s sensory observation. What should be at stake in the debate between internalists and externalists is the status of the ‘guess’ that the organism embodies; namely, the *posterior beliefs*^6^ encoded by internal states, and whether this guess does, or does not constitute the limit of ‘cognition’, understood as the avoidance of surprisal, or informational homeostasis.

The *posterior belief* (i.e., recognition density) represents the system’s ‘best guess’ regarding the causes of its sensory states, and is embodied or encoded by the states of the organism; technically, internal states of the Markov blanket (Friston 2012). Under the FEP, the *system’s* posterior belief is refined or ‘tuned’ under the generative model, through a process of variational (approximate Bayesian) inference, and becomes a tight bound on the *true* posterior belief it aspires to (Friston et al. 2016b).

The generative model is a statistical construct that transcribes the expected sensory causal regularities in the process generating sensory states. The generative model is used to model the set of viable phenotypical statistical relations (preferences, and action policies) that must be brought forth by the organism in active inference: in short, a model of a viable state of being for the organism. Through active inference, internal states are tuned and this tuning changes its posterior belief, and hence the organism’s ‘best guess’ about what caused its sensations (that usually include its own actions). In other words, a generative model can be used to understand how organisms are able to track (infer) their own behaviour.

The FEP is based on the idea that the functions and changes in the structure of living systems conform to approximate Bayesian inference. This assumption rests on the claim according to which living systems avoid surprise (cf. Sect. 2.); approximate Bayesian inference under the FEP, then, is just one sensible strategy to understand how living systems avoid surprise and the dispersion of their sensory states. It rests on what we described earlier as the generative model (the control system), the recognition density (the living system), and the generative process (the external world, which includes the organism’s actions). Simply put, the relation between these is that the recognition density changes as a function of the control system; and because the control system constitutes expectations about the world conditioned upon the preferences of the living system, the living system turns out to change so as to become (statistically) consistent with the preferred world; that is, according to its preferences and expectations about the world.

Under the FEP, ‘cognition’ is what the recognition density, or living system does (i.e., changing to elude surprises and maintaining informational homeostasis by minimizing free energy), and the way one studies cognition (i.e., what the system does), is by developing, simulating, and analysing the possible generative models that explain how the recognition density of interest (the system of interest) changes so as to attain minimal free energy.

In other words, ‘drawing the bounds cognition’ means defining the *recognition density* of the system of interest, and identifying a *generative* model that explains changes in that system that follow variational Bayesian inference.^7^ In this sense, cognitive science might be understood as the study of generative models and processes: it is in the business of modelling the correlational or causal structure of actions and observations of the organism. The generative model, then, is not the *vehicle* of something like content or mutual information; instead, it is the tool that we use to study cognitive systems (as explanatory model), and indeed, perhaps more speculatively, the guide, or path living systems entail and follow to stay alive (as control systems). The vehicle is the recognition density (also called the variational density), the ‘best guess’ that the system of interest embodies, and whose function and structure can be studied using the generative model.

This means that we can study cognition meaningfully as it occurs in individuated systems at the respective scales at which those systems exist; e.g., the brain in ontogeny, or large-scale ensembles like species over phylogenetic time. Since organization at each level depends upon the integration to the entire dynamics, one can also study cognition ‘across boundaries’. Below, we will see that we can formalise how the system moves from one state to the other in terms of a free energy bounding dynamics. This dynamics integrates systems of systems; all individuated as nested Markov blankets of Markov blankets.

In summary, we are suggesting that organisms use a statistical trick—i.e., the minimization of variational free energy—to track the causes of their sensory states and to select appropriate actions. The key is to note that organisms are organized such that they instantiate the prior that their actions will minimise free energy. This mechanics of belief is the only causally relevant aspect of the variational free energy. The free energy may or may not exist; what is at stake is the causal consequences of the *action*-*guiding beliefs of organisms and groups of organisms*, which are harnessed and finessed in the *generative model* (Ramstead et al. 2019). What matters is that organisms are organized such that they instantiate such a prior to guide their action.

### Enactivism 2.0

The generative model, as we have seen, functions as a *control system*. That is, its function for the cognitive system is to generate of adaptive patterns of behaviour. In the parlance of the FEP, its purpose is to guide the evaluation and selection of *relevant action policies* (Friston et al. 2016b). The generative model is a strange beast in the variational framework, in that it exists only insofar as it underwrites the organism’s inference about states of affairs and subsequent action selection. Since the free energy expected following an action, which determines the policy to be selected, is defined in terms of the generative model, the latter is the cornerstone of the self-evidencing process.

This emphasis on adaptive action aligns active inference with one brand of radical accounts of cognition, namely *enactivism*. Indeed, it has been argued that the FEP provides an implementation of enactivism, and in a sense supersedes or absorbs classical (i.e., autopoietic) formulations of enactivism (i.e., Froese and Di Paolo 2011; Thompson 2010; see Kirchhoff 2018a; Kirchhoff and Froese 2017, for a detailed argument to this effect). Active inference is inherently a pragmatist or enactive formulation, and can be contrasted with non-enactive appeals to Bayesian principles of cognition, such as predictive coding.

However, because it relies fundamentally on formulations from information theory, active inference is in tension with a few of the more (arguably) conservative elements of the enactive theory. Indeed, classical enactivism has typically rejected the appeal to information theory to describe cognition (e.g., Thompson 2010). We believe this is a hangover from another age in cognitive science. And, more to the point, this conservatism has not prevented the proponents of active inference from taking up the banner of enactivism (Bruineberg et al. 2016; Engel et al. 2016; Kirchhoff and Robertson 2018; Ramstead et al. 2018a). Active inference provides a theoretical model for enactment. (Allen 2018) has called this form of enactivism, based in information theory, ‘enactivism 2.0’, or Bayesian enactivism.

### Nestedness: or how to study cognition beyond the brain

**Fig. 5:**
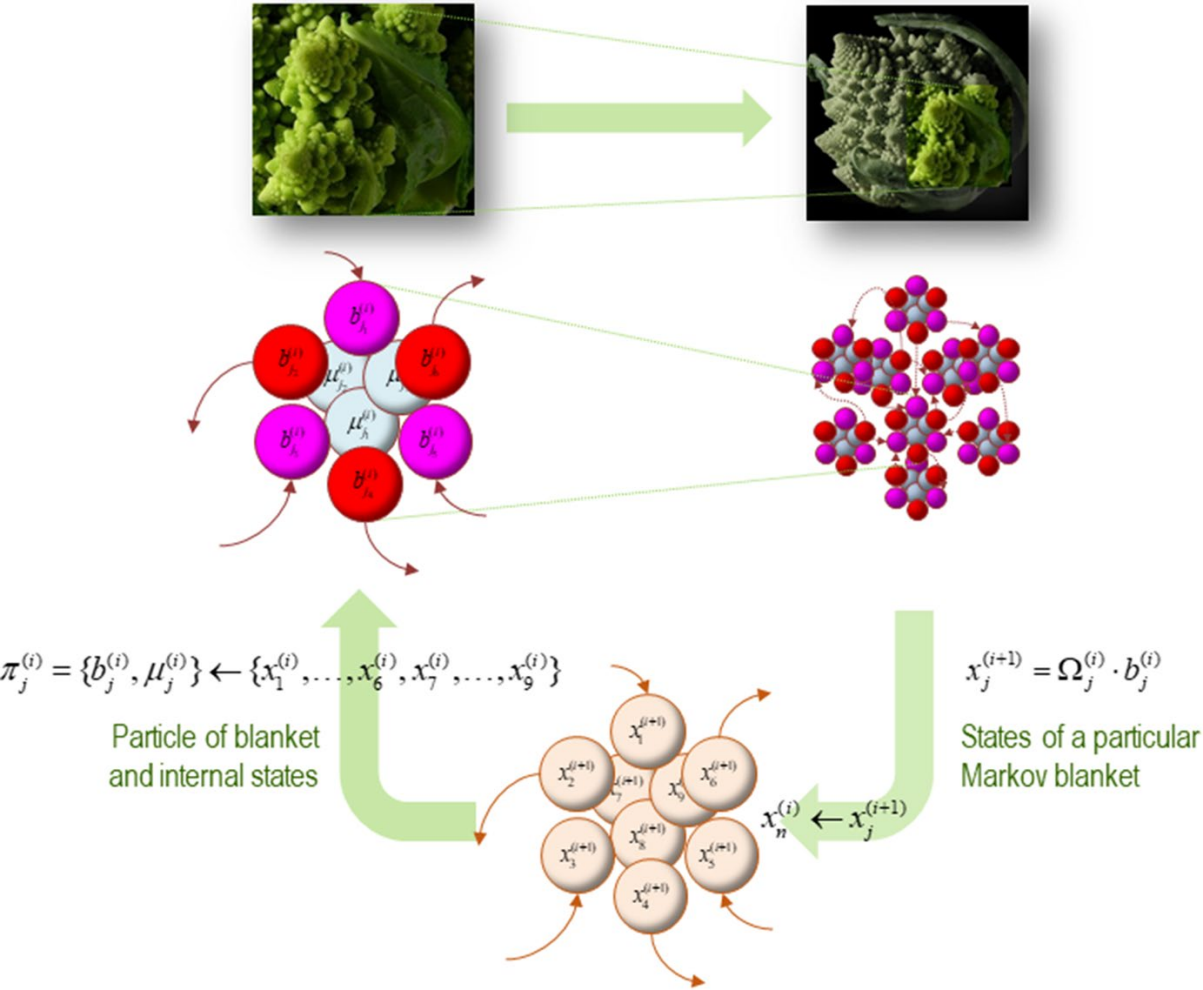
Blankets of blankets. This figure depicts the recursively nested structure of Markov blankets that forms the basis of our formal ontology of cognitive systems. In this scheme, successively larger and slower scale dynamics arise from, and constrain, those dynamics arising at subordinate (smaller and faster) scales. Consider an ensemble of vector states (here, in the lower panel, nine such states are depicted). The conditional dependencies between these vector states define a particular partition of the system into *particles* (upper panels). The effect of this partition into particles is, in turn, to partition each of these particles into blanket states and internal states. Blanket states comprise active states (red) and sensory states (magenta). Given this new partition, we can summarize the behaviour of each particle in terms of (slow) eigen modes or mixtures of its blanket states, which in turn produces vector states at the next (higher) scale. These constitute an ensemble of vector states and the process can begin anew. The upper panels depict this bipartition into active and sensory states for a single particle (left panel) and for an ensemble of particles. The insets at the top of the figure illustrate the self-similarity that arises as we move from one scale to the next. In this figure, Ω·*b* denotes a linear mixture of blanket states that decay sufficiently slowly to contribute to the dynamics at the level above. Adapted from Ramstead et al. (2018a). (Color figure online)

The existence of Markov blankets at one scale means that interaction amongst components at that scale are mediated by states belonging to their respective Markov blankets. These active exchanges have a sparsity structure that induces *nested sets of Markov blankets*—that is, Markov blankets of Markov blankets (Kirchhoff et al. 2018; Ramstead et al. 2018a). The central idea behind the multiscale integration of Markov blankets is that the particular statistical form and the specific partitioning rule that governs the Markov blanket allows for the assembly for larger and larger Markov blankets (of cells, of organs, of organisms, of environments, and so on). This is because Markov blankets at increasingly larger scales of systemic organisation recapitulate the statistical form of Markov blankets at smaller microscopic scales of systemic organisation. This can be shown to follow from the observation that any meaningful statistical separation between internal and external states at the scale of, for example, complex organisms, a macroscale Markov blanket must be present, whose sensory and active states, distinguish this organism from its local niche, and which itself is composed of smaller and smaller Markov blankets sharing the same statistical form as the macroscopic Markov blanket (see Fig. 5).

Figure 5 illustrates the idea of Markov blanket formation at any scale of hierarchical and systemic organisation, thus speaking to the notion that organisms and their local environment will be “defined not by a singular Markov blanket, but by a near-infinite regress of causally interacting Markov blankets within Markov blankets.” (Allen and Friston 2018, p. 19). This, in turn, provides an integrated perspective from which to approach the multiple scales of self-organisation in living systems.

The multiscale partition of model parameters, encoded by internal states of the Markov blanket, attunes itself to the sufficient statistics of the generative process that generated the sensory observations, tuning its internal states by bounding free energy. This process occurs at and across spatiotemporal scales, effectively integrating the system through dynamics. Indeed, for each system individuated at a given scale, one can define a generative model entailed by the dynamics at the scale above; which speaks to the complementarity between specialisation and statistical segregation, on the other hand, and functional integration, on the other (Badcock et al. 2019).

**Fig. 6:**
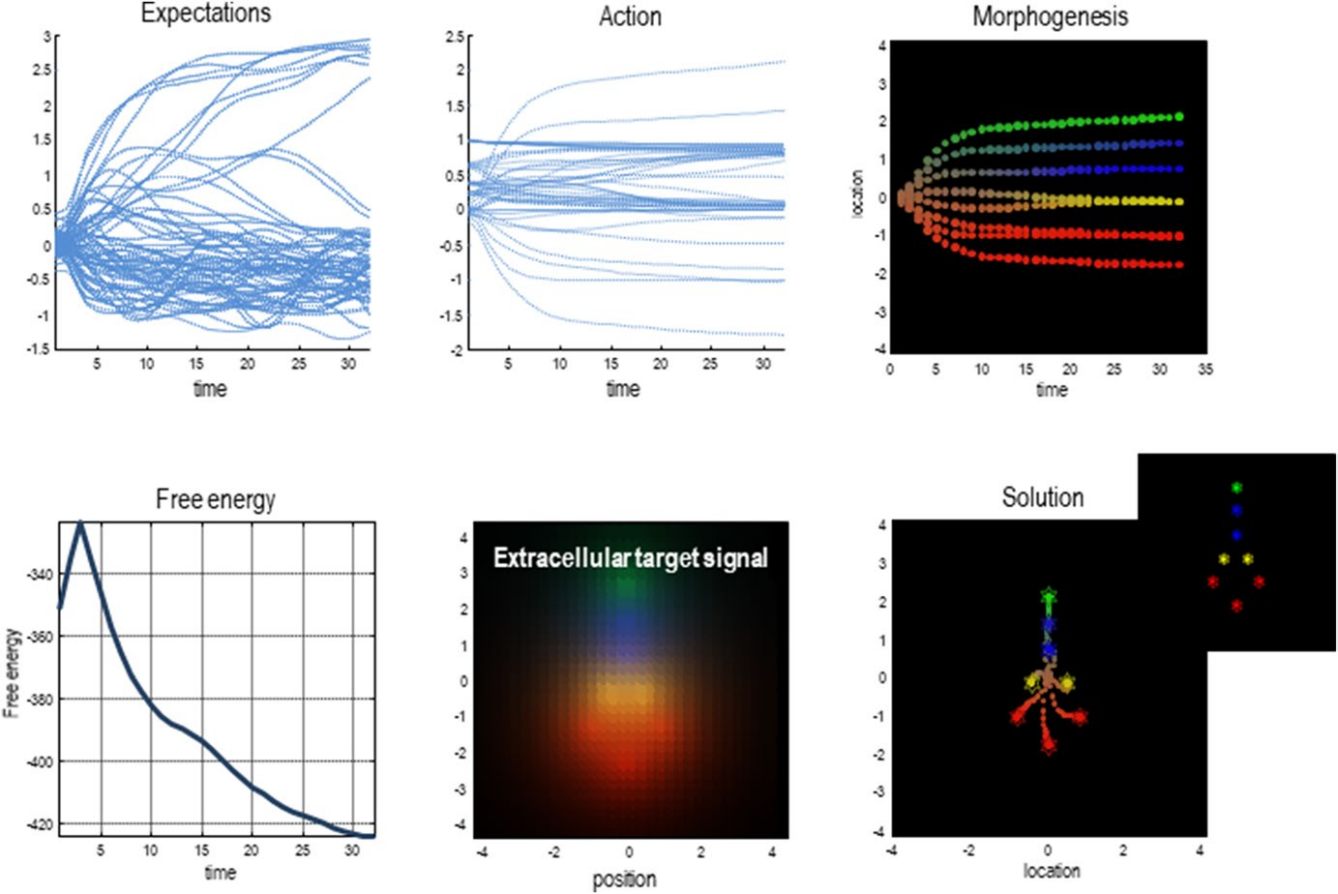
Multiscale self-organization and active inference. This figure depicts variational free energy being minimised across scales through active inference. It presents the results from a simulation of morphogenesis using the active inference framework (Friston et al. 2015). The simulation used a gradient descent on variational free energy to simulate a group of cells self-assembling into a larger pattern (i.e., target morphology). The simulation employed an ensemble of eight cells. Each cell was equipped with the same generative model, which is a metaphor for shared genetic information. This generative model generated a prediction of what each cell would sense and signal to other cells (chemotactically) for any given location in a target morphology. In other words, the model predicted what each cell would expect to sense and signal if it were in that location (lower middle panel—extracellular target signal). Each cell engaged in active inference, by actively moving around to infer its place in the target morphology relative to other cells. In doing so, each cell minimised its own variational free energy (and by proxy, its surprise or self-information). Remarkably, the fact that all cells shared the same generative model allowed their individual active inference to minimise the free energy of the ensemble, which exists at the scale above the individual cells. Each of the cells that make up the ensemble shares the same generative model. Crucially, the sensory evidence for the model with which each cell is equipped is generated by another cell. The arrangement that minimises the free energy of the ensemble is the target morphology. This means that each cell has to ‘find its place’; the configuration in which they all have found their place is the one where each cell minimises its own surprise about the signals it senses (because it knows its place), and in which the ensemble minimises the total free energy as well. The upper panels show the time courses of expectations about the place of each cell in the target morphology (upper left), as well as the associated active states that mediate cell migration and signal expression (upper middle). The resulting trajectories have been projected onto the first (vertical) direction and color-coded to show cell differentiation (upper right). The trajectories of each individual cell progressively, and collectively, minimize total free energy of the entire ensemble (lower left panel), which illustrates the minimization of free energy across scales. The lower right panel shows the configuration that results from active inference. Here, the trajectory is shown in small circles (for each time step). The insert corresponds to the target configuration. In short, all multiscale ensembles that are able to endure over time must destroy free energy gradients, which integrates system dynamics within and between scales. Adapted from Friston et al. (2015). (Color figure online)

Free energy is an additive or extensive quantity minimised by a multiscale dynamics integrating the entire system across its spatiotemporal partitions (Ramstead et al. 2019). There is also, therefore, only one free energy for the entire system, which is simply the sum of free energies at all the relevant scales (see Fig. 6). The whole system dynamics leverage internal states across temporal and spatial scales, to integrate the system across scales. This means that the variational approach accommodates both multiscale partition of the recognition density, and a multiscale integration (through active inference).

The underlying philosophical point is that states that are statistically isolated by Markov boundaries become integrated under one dynamics in active inference; they come to parameterise one generative model (the one entailed by the adaptive behaviour of the whole system), thereby guiding one integrated action across temporal and spatial scales. Internal states that are inferentially secluded at one scale become absorbed into higher order Markov blankets and dynamics at the scale above. This means that the epistemic seclusion of internalism is, in a sense, illusory or partial; since the entire organism engages in active inference across scales. Under the FEP, inferential seclusion coexists with existential (pragmatic) integration through dynamics (i.e., adaptive behaviour).

This perspective vindicates an integrationist ontology of the boundaries of cognition, while retaining the possibility of granting epistemic priority to any of these boundaries, given explanatory interests. The nested Markov blankets perspective answers the question of how to understand the generative model from this multiscale perspective. The challenge, now, is to develop the theoretical apparatus to describe the boundaries of cognition at higher scales. We address this issue in the next subsection.

In a nutshell, at any scale, the relevant Markov-blanketed systems are composed of parts that, in virtue of their (relative) conditional independence, can also be described as Markov blanketed systems. Each of these separate Markov blanketed subsystems might count as separate systems, i.e., one cognitive subsystem can be a nested part of another system. However, all these nested boundaries are integrated within the same system. More precisely, all the subsystems that are individuated by their own Markov blanket are *integrated* as one single dynamical system through the *system dynamics* (i.e., *adaptive action*). Collectively, there is only one (hierarchical) generative model and therefore one free energy functional, for the ensemble of nested blankets (where each constituent blanket itself has a generative model and accompanying free energy functional). This sort of nesting is particularly prescient for hierarchical systems like the brain. In this brain-bound setting, the integrated Markov blanket could be regarded as comprising the brain’s sensory epithelia and motor (or autonomic) efferents, while internally nested Markov blankets are a necessary feature of neuronal (e.g., cortical) hierarchies (Shipp 2016; Zeki 2005; Zeki and Shipp 1988). At each and every level of the cortical hierarchy the associated free energy is minimised by neuronal dynamics, such that the total free energy of the brain is upper-bounded in accord with the FEP.

### Multiplicity: or how to describe cognition beyond the brain

Central to our discussion is the concept of joint phenotype, which we have introduced in Sect. 1 in terms of repertoire of highly probable states and traits. Some of those states are contained within the organism (e.g., brain states), and other traits extend far beyond the internal states of an organism (e.g., states of the niche). We use the concept of joint phenotype to support our description of the boundaries of cognition at higher scales.

Typically, joint phenotypes are seen as shared ‘extended phenotypes’ (Dawkins 1982). Extended phenotypes are traits (e.g., niche construction outcomes like beaver dams) that, like physiological states, undergo selection due to their fitness enhancing impact. In the case of an extended trait, the impact is on the genes having favoured the reproduction of that extended trait (e.g., beavers’ genetic disposition to build dams).

Extended phenotypes, therefore, are extensions from genes to the extended trait. Accordingly, the typical view of the joint phenotype broadly construed describes coextensive phenotypic traits consistent with two or more different species’ genetic makeup. In that case, all parties can be ‘joint owners’ of the trait; for instance, the insect and the plant are joint owners of *the portion of the leaf* eaten by the insect (Queller 2014).

The FEP interpretation of the joint phenotype that interests us here brings this a step further. On that view, coextensive phenotypic traits *do not* need to be included in the extended phenotype. They can include biotic or abiotic traits, like ecological cascades produced by niche construction, or other ‘seemingly’ random effects of organismic activity. These are not directly related to the genetic makeup of either party, while nonetheless being seen as having a systematic and evolutionary significant impact on fitness.

With the FEP, one can study organism-niche complementarity that obtains through phenotypic accommodation and niche construction over development (i.e., adaptation) using variational free energy (Bruineberg et al. 2018; Constant et al. 2018b), and thereby predict the influence of a trait on fitness. Hence, one can conceive of and study joint phenotypic traits as non-genetically specified traits by studying the changes in the statistical relationship that bounds those traits to the states of the organism(s).

Now, the point we want to motivate here is that—especially in humans—many traits of the constructed niche defining the human joint phenotype increase state-trait complementarity by smoothing the attunement process, or variational free energy minimising process. For instance, in developmental psychology and niche construction theory, it is argued that the material artefacts populating human niches enable individuals to deal with perceptual uncertainty (Christopoulos and Tobler 2016; Dissanayake 2009) by constraining, and directing sensory fluctuations in their surrounding (Constant et al. 2018a).

Briefly, computing expected free energy requires computing the cost of a policy (where the cost is given in terms of the divergence between posterior beliefs and preference about sensory outcomes), and the expected ambiguity, or expected ‘certainty’ about the sensory outcome relative to one’s beliefs about the state of the world (i.e., expected surprise) (see Friston et al. 2016a, b for a detailed treatment). Artefacts that populate human niches can be seen as doing much of the legwork in computing the expected ambiguity term that constitute expected free energy. In that sense, they ease the modelling activity of the organism, understood as expected variational free energy minimization (Constant et al. 2018b); cf. epistemic affordance (Parr and Friston 2017).

Thus, especially in humans, when taking the FEP perspective, one can include external, joint phenotypic traits within the boundaries of cognition for higher scales systems like joint phenotypes, or bodies–environment systems. It also means that under the FEP, one could meaningfully study cognition ‘from outside the brain’, for instance, by producing a generative model of an higher scale system (e.g., that of the leaf-insect system) and by simulating the effects of external factors on variational free energy, like environmental cues (Sutton 2007); cultural practices (Vygotsky 1978); ecological information (Gibson 1979). Again, this speaks to the idea that the relevant boundaries of cognitive systems are relative to explanatory interests (e.g., cognition from the point of view of neurophysiology for cognitive neuroscientists, or cognition from the point of view of ecology, for behavioural ecologists).

The Markov blanket formalism might allow us to study the transient assembly of cognitive boundaries over time, in the spirit of the models considered above. Indeed, the original simulation studies employing the Markov blanket formalism were about the carving out of Markov boundaries by the dynamics of free energy minimization (Friston 2013). The variational framework, then, might allow us to model how organisms extend their Markov blankets into the environment, at a host of different spatial and temporal scales (Ramstead et al. 2019); e.g., to model the spider’s web extending its ensemble of sensory states to include states outside its body (Kirchhoff and Froese 2017).

In summary, the boundaries of cognitive systems are *nested* in that any system is made up of components, which (given that they, too, exist in a minimal sense) have a boundary that can be formalised as a Markov blanket. A given organism is essentially a hierarchical set of nested Markov blankets. Furthermore, there is a hierarchical listing of scales, in the sense that every state at a given scale is itself a mixture of blanket states at a smaller scale (see Figs. 5, 6). The subsystems of interest here range from intra-cellular blanketed systems (e.g., organelles) to the blanket of the entire species. By the very fact that they are nested in this way, up to the scale of the species, the boundaries of any cognitive process of cognitive dynamics are *multiple*, in the sense that cognitive systems at different scales are integrated in one multiscale cognitive dynamics. The boundaries and scale that are *relevant* will depend on the kind of investigation we are aiming at, the phenomenon that is of interest, etc.

## Concluding remarks: towards multidisciplinary research heuristics for cognitive science

In this paper, we have attempted to overcome a common tendency to think of the boundaries of cognitive systems as either brain-based, embodied, or ecological/environmental by appealing to a multiscale interpretation of Markov blankets under the variational FEP. The resulting *multiscale integrationist* perspective suggests that the boundaries of cognition are multiple and nested.

Some of the radical externalist views on cognition that we have discussed suggest that the divide between internalism and externalism is problematic (Thompson and Stapleton 2009). We agree, precisely because each of these two options begs the question over where to look for the realisers of cognition. We argued in favour of an *ontological pluralism* based on a multiscale formulation of Markov blankets under the FEP. We argued that ontologically, states statistically insular or segregated at one scale are integrated by the dynamics (i.e., adaptive behaviour) at scales above. States separated by their respective Markov blankets are dynamically and statistically linked as states of the same higher-order system. The recursively nested, multilevel formulations of the Markov blanket formalism under the FEP allow to study the realisers of cognition, while acknowledging that they are a moving target; they shift according to the level of inquiry.

Some radical externalist views, enactive approaches especially, cast cognition as a relational phenomenon that equally recruits states of the brain, the body, and the world. The view we propose here agrees with the relational aspect of this project, but rejects the a priori emphasis in the assumption that all factors contribute equality to the causal patterns of interest. That cognition is inherently relational, that it integrates the contribution of states that are internal (systemic) and external to any given boundary, does not imply that the contributions of all relevant components are equal. Certain kinds of cognition rely mainly on the contributions of internal states (e.g., mental calculations); other activities are more embodied, and rely mainly on physiological or morphological states (e.g., walking); and other still depend most on the influence of abiotic, environmental factors or culturally patterned practices (e.g., driving a car). The approach we advocate here casts cognition as radically relational at each scale, even within the brain; e.g., relations between cells, relations to the brain’s microenvironment, between different networks or again, between different patterns of functionally integrated units; without for all that endorsing the view that nothing matters more than anything else. This speaks to the necessity of *methodological pluralism* in cognitive science; and to the importance of developing new interdisciplinary research heuristics to determine and study, for any phenomenon, the relevant levels of description that are necessary to account for it.

Our multiscale integrationist formulation of the boundaries of cognition rejects any kind of essentialism about the boundaries of cognition. It suggests that explanations of cognition will differ conditioned on the phenomenon and our explanatory interests. In this sense we are more aligned with (Clark 2008) when he encourages us to “let a thousand flowers bloom” (p. 117). However, we restrict the scope of this gardening project by arguing that the FEP plays a coordinating and constraining role on the kind of explanations one should be looking for in the cognitive sciences.

## Footnotes

1 We do not intend this list of relevant disciplines to be exhaustive.

2 Note that we will use the term ‘belief’ throughout to mean ‘probabilistic Bayesian belief’, which is a probability distribution encoded by states of the organism. A belief in this sense need not have any content; it is *not* a belief in the traditional philosophical sense, but instead should be read as synonymous with ‘probability distribution’.

3 A very similar conflation of epistemological and ontological notions of a ‘model’ was apparent at the inception of cybernetics in the form of the Good Regulator Theorem (every system that regulates its environment must be a good model of that environment) (Conant and Ashby 1970). The free energy principle formalises this notion by equipping existential dynamics with an epistemological corollary cast in terms of inference.

4 Also known as approximate Bayesian inference. Variational or approximate Bayesian inference necessarily entails the minimisation of variational free energy, under a generative model and an assumed form for posterior beliefs.

5 This ‘aboutness’ is a key aspect of the FEP and follows from the fact that internal states encode or parameterise a probability distribution over external states. In other words, internal states are the sufficient statistics of Bayesian beliefs (about external states). See (Ramstead et al. 2018a), Box 4.

6 By definition, the posterior belief encoded by internal states approximates the true posterior belief when free energy is minimised. It is often referred to as a recognition density or approximate posterior belief.

7 This is especially true in specialised fields such as computational psychiatry, where a crucial part of the generative model—namely prior beliefs—completely specify behaviours and preferences. This means that any behavioural phenotype can be, in principle, quantified or understood in terms of prior beliefs. See (Schwartenbeck and Friston 2016) for a worked example.
